# Efficacy and Tolerability of Clarema 1% Cream and Hirudoid 40000 U.APTT Gel in the Topical Treatment of Haematomas and/or Subcutaneous Haematic Extravasations

**DOI:** 10.5402/2012/504151

**Published:** 2012-01-17

**Authors:** Tiziana Polieri, Enrico Orsoni, Giorgio Saponati, Enrico Castellacci

**Affiliations:** Sport Medicine and Traumatology Unit, Lucca General Hospital, 55100 Lucca, Italy

## Abstract

Ninety-six caucasian both-gender patients with haematomas and/or subcutaneous haematic extravasation of traumatic or surgical origin were randomized to receive local treatment (max 10 days) with heparan sulfate cream or glycosaminoglycan-polysulphate (GAGPS) gel. Signs (oedema, disability, and colour of the lesion) and symptoms (pain at rest and at movement) (scored 0–3), the sum of the scores (primary end point), and the size of the lesion were evaluated at the baseline visit and afterwards every 5 days. The rate of the patients completely healed at the end of the study was also recorded. The results of the study showed that heparan sulfate 1% cream was comparable or superior to GAGPS gel in relieving signs and symptoms. No AEs were recorded.

## 1. Introduction

Heparan sulfate is a mucopolysaccaride present in the arterial and venous wall, provided by fibrinolytic and anticoagulant activities. Heparan sulfate is a member of the glycosaminoglycan family of carbohydrates, and its structure is very closely related to heparin, both consisting of a variable sulfated repeating disaccharide units. The most common disaccharide unit of heparan sulfate is a glucuronic acid linked to N-acetylglucosamine, typically making around 50% of the total disaccharide units [1–3]. In pharmacodynamic studies, heparan sulfate was shown to inhibit thrombogenesis and to promote the fibrinolytic process through both the intrinsic and the extrinsic pathway. The mechanism of action includes alterations of the other steps of the fibrinolytic process by activating the proactivants and antagonising the plasmin inhibitors, thus showing an anti-Xa and anticomplement activity. Given by oral route in patients with chronic venous insufficiency, heparan sulfate proved to be effective in reducing symptoms of itching, oedema, spontaneous pain, and nocturnal cramp, while exerting marked profibrinolytic and prohemorheological effect [4, 5]. A cream formulation of heparan sulfate (SPC Clarema 1% cream) has been obtained by means of specific methods of extraction and purification. This formulation exerts marked antithrombotic properties, which are the result of an intensive profibrinolytic activity and of the activation of antithrombin III (AT III). Heparan sulfate in cream form, when applied in subjects with alteration of the superficial venous circulation, gave clinically significant results in terms of pain remission, and reduction of the local oedema and of phlogistic infiltration. Heparan sulfate 1% cream is actually indicated for the local treatment of sequelae of phlebothrombosis, thrombophlebitis, venous ectasias of lower limbs, cutaneous affections of vascular origin (superficial periphlebitis, inflammatory oedema, etc.), haematomas of traumatic origin, and postphlebitis disease of lower limbs. This study was aimed at obtaining further information on the clinical use of the heparan sulfate 1% cream formulation in the treatment of subjective symptoms and objective signs of haematomas and subcutaneous haematic extravasations of traumatic/surgical origin. The effects of heparan sulfate 1% cream were compared with those of glycosaminoglycan-polysulfate (GAGPS) gel, a heparin-like antithrombotic agent indicated in the treatment of postphlebitic symptoms and haematomas.

## 2. Patients and Methods

### 2.1. Patients

Patients of both genders aged 18 to 75 years with an evidence of a posttraumatic or postsurgical haematoma and/or haematic extravasation started no more than 3 days earlier were enrolled. To be eligible for the study, the lesion was required to have a size in the range 2 × 2 cm (approximately 4 cm^2^) to 20 × 20 cm (approximately 400 cm^2^); in the case of multiple lesions, the largest satisfying the inclusion criteria was taken into account. Patients were enrolled if they had at least two subjective symptoms/objective signs of haematoma and/or haematic extravasation of at least moderate degree (i.e., with score 2). Patients had to be excluded from study participation in presence of history or current evidence of coagulation disorders; treatment with anticoagulant, fibrinolytic, antiplatelet or hemorheologic agents, or with drugs potentially acting on coagulation or platelet aggregation parameters; treatment with steroidal and nonsteroidal anti-inflammatory drugs (NSAIDs). Patients were also excluded if they had history or evidence of important medical conditions such as cardiovascular diseases (e.g., congestive heart failure NYHA class >1, coronary artery disease, myocardial infarction, severe hypertension, cardiac arrhythmias), liver (i.e., AST/ALT higher than twice the upper limit of normal range) or renal (i.e., creatinine >2 mg/dL) insufficiency, metabolic or endocrine diseases (e.g., uncontrolled diabetes mellitus), and any other underlying medical condition that could interfere with the study evaluation parameters (immunocompromised patients, evidence of cutaneous lesions, such as wounds, ulcers, sores, etc., or other skin diseases that impaired the skin integrity of the surface selected for treatment; evidence of serious concomitant lesions of the surface selected for treatment, such as fractures, tendon ruptures, etc.; need of additional measures for the management of the traumatic lesions, such as immobilisation with plaster or other measures, surgical procedures, etc.).

### 2.2. Study Design and Treatments

The study was performed according to an open-label, controlled, randomized, single-centre, parallel design. The selection of an open-label design was due to the fact that different pharmaceutical forms (cream and gel) were used in the study. During the baseline visit, patients eligible for the study were randomly assigned to receive one of the following treatments: heparan sulphate 1% cream (Clarema, Farmaceutici Damor S.p.A., Italy) or glycosaminoglycan-polysulphate (GAGPS) gel (Hirudoid 40000 U.APTT, Sankyo Pharma Italia S.p.A., Italy). The study drugs were applied three times daily, starting from the baseline visit, in an amount adequate for the size of the lesion. Follow-up visits were scheduled at 5-day intervals of therapy, for a maximal observational period of 10 days and a maximum of three visits in total. Treatment with any other drugs potentially acting on coagulation or platelet aggregation was not permitted as well as steroidal and nonsteroidal anti-inflammatory drugs (NSAIDs) other than paracetamol. Nonpharmacological measures for the management of the traumatic lesions, such as immobilization with plaster or other measures, surgical procedures, ultrasounds, laser therapy, and so forth, were not allowed. The use of paracetamol (500 mg tablets) was allowed as rescue analgesic medication for the entire study duration, as well as contact ice or cold application.

### 2.3. Outcome Measures

The change from baseline of sum of scores of subjective symptoms and objective signs (pain at rest, pain at movement, oedema, functional disability, and colour of the lesion) was the primary efficacy variable. Signs and symptoms were measured using a 0–3-point rating scale (absent, mild, moderate, and severe). The secondary efficacy variables were the changes from baseline of each individual subjective symptom and objective sign; the rate of patients with complete healing at the end of treatment (i.e., score of all signs and symptoms equal to 0); the changes from baseline of the size of the lesion (largest and lowest diameters, and their product) and the use of relief paracetamol (rate of users and number of used tablets). Local (at the site of the lesion) and general adverse events (AEs) were the safety variables in this study.

### 2.4. Ethics

Informed consent was signed by all participants prior to the start of any study-related procedure. The study protocol was approved by the reference Ethic Committee of the study site.

### 2.5. Statistical Analysis

The sample size was based on a hypothesis of noninferiority between treatment groups on changes from baseline to day 10 (or early withdrawal) in the primary efficacy variable. The limit for noninferiority was defined as the lower limit of a two-sided 95% confidence interval (CI) for the difference between least square means (LSMs) of sum of score of symptoms and signs being −0.2 or above. This limit had been chosen taking into account that it was expected that the haematoma/extravasation was not completely healed at the final visit in most of patients and, therefore, signs/symptoms of at least mild intensity (although improved versus baseline) were still present at the final visit. Estimating a standard deviation of 0.30, a total of 96 evaluable patients (48 in each group) was expected to provide approximately 90% power for fulfilling the above hypothesis. The following populations were considered for data analysis: safety, that is, all randomised patients who received at least one application of study medication; intention to treat (ITT), that is, all patients of the safety population without major violations of study procedures and who were visited at least once after the baseline visit; per protocol (PP), that is, all patients of the ITT population who completed the study. For the analysis within group, the 95% CI for the mean changes from baseline was calculated. The assessment of noninferiority between the two groups with regard to primary efficacy variable was performed by calculating the bilateral 95% CI for the difference between LSMs from an analysis of covariance (ANCOVA) model, which included treatment as factor and baseline as a covariate. The comparisons between groups of the changes from baseline of individual symptoms and signs, and of the size of the lesion (largest and lowest diameters, and their product) were performed as for the primary variable, without testing for noninferiority. The comparisons between groups of the rates of complete healing at the end of treatment and of the rate of patients requiring the use of relief paracetamol were performed by means of Chi-square test or Fisher' exact test. The comparison between groups of the total amount of used tablets of paracetamol in the entire study duration was performed by means of unpaired Student' *t*-test. Missing data were replaced according to Last Observation Carried Out (LOCF) method. General AEs were to be coded as system organ class (SOC) and preferred term using MedDRA dictionary version 7.1. However, no adverse events were reported in any patient in both groups.

## 3. Results and Discussion

### 3.1. Patient Disposition and Baseline Characteristics

A total of 96 caucasians patients were randomized to receive treatment with heparan sulfate cream or glycosaminoglycan-polysulphate (GAGPS) gel. All patients concluded the study according to the protocol, therefore the safety, the ITT, and the PP populations coincided.  Table 1 shows the demographic and baseline clinical characteristics in the two groups. There were no statistically significant differences between groups for any of the measured demographic and baseline clinical parameters.

### 3.2. Efficacy

**Primary Efficacy Variable**

**Figure 1:**
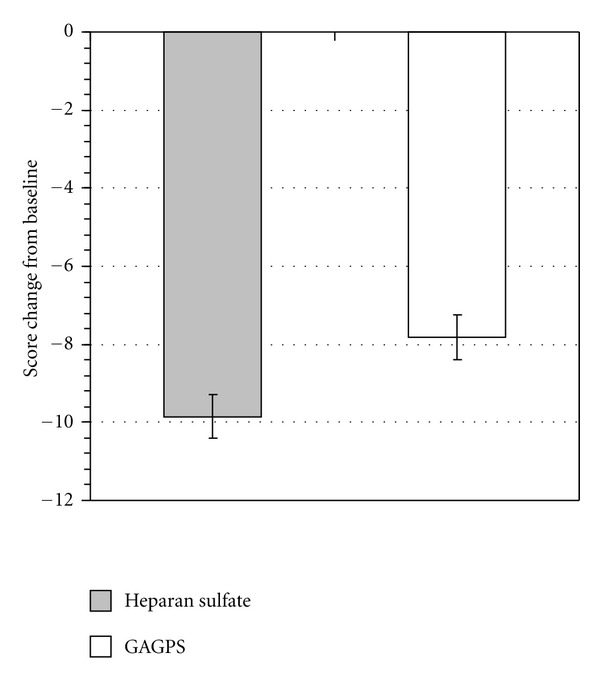
Primary efficacy variable. Least square means of score variation of all symptoms and signs (mean values and 95% CI).

The mean change from baseline to endpoint of the sum of the scores was higher in the heparan sulfate group (−9.8; 95% CI: −10.4 to −9.2) than in the GAGPS group (−7.9; 95% CI: −8.6 to −7.1) (Figure 1). The difference between the adjusted means of the heparan sulfate group (−9.85) and of the GAGPS group (−7.82) was equal to 2.03. The 95% bilateral CI for the difference between the adjusted means of the heparan sulfate group and the GAGPS group in the ANCOVA model was 1.23 to 2.82, and the lower limit was greater than the prespecified limit of −0.2, thus showing that heparan sulfate was noninferior to GAGPS. The difference between groups was statistically significant, clinically in favour of the heparan sulfate group.

**Secondary Efficacy Variables**

The rate of patients with complete healing was significantly (Fisher's exact test: *P* < 0.0001) higher in the heparan sulfate group (25 patients, 52.1%) than in the GAGPS group (4 patients, 8.3%). The results for individual subjective symptoms and objective signs changes are reported in Table 2. For each of the secondary variables, the mean decrease from baseline to endpoint is reported together with the differences between the adjusted means of the heparan sulfate and GAGPS groups. The rate of patients with complete disappearance for each symptoms/signs of haematomas is also reported. The 95% bilateral CIs for the differences between the adjusted means of the heparan sulfate and the GAGPS group in the ANCOVA model show that the difference between groups was statistically significant for all the clinical parameters evaluated, except for the pain at rest.

### 3.3. Use of Paracetamol

The number and rate of patients who took paracetamol were higher in the heparan sulfate group (14 patients, 29.2%) than in the GAGPS group (5 patients, 10.4%). The difference between groups was statistically significant (*P* = 0.0386). At Visit 2, the mean number of used paracetamol tablets was higher in the heparan sulfate group (1.1; 95% CI: 0.4 to 1.8) than in the GAGPS group (0.5; 95% CI: 0.0 to 0.9), while, at Visit 3, the mean number of used paracetamol tablets was small and comparable in the heparan sulfate group (0.3; 95% CI: −0.2 to 0.7) and in the GAGPS group (0.4; 95% CI: −0.0 to 0.8).

The mean number of used paracetamol tablets during the entire study was higher in the heparan sulfate group (1.4; 95% CI: 0.4 to 2.4) than in the GAGPS group (0.9; 95% CI: 0.0 to 1.7); however, the difference between groups was not statistically significant.

### 3.4. Safety

There were no adverse events reported in any patient in both groups.

## 4. Conclusions

The objective of this controlled randomized study was to compare the efficacy and the tolerability of heparan sulfate 1% cream and glycosaminoglycan-polysulfate (GAGPS) gel in patients with haematomas and/or subcutaneous haematic extravasations of traumatic or surgical origin. GAGPs is a heparin-like antithrombotic agent widely used in Italy for the treatment of superficial phlebitis, postphlebitic symptoms, and haematomas. The results showed that heparan sulfate 1% cream was comparable or superior to GAGPS gel in the relief of sum of scores of signs and symptoms (primary efficacy variable) and treatment with both study drugs was associated with significant improvements of all individual subjective symptoms and objective signs. The improvements from baseline were significantly more marked in the heparan sulfate group than in the GAGPS group for the primary efficacy variable and for all the individual subjective symptoms and objective signs (apart from pain at rest) as confirmed by the mean decrease from baseline to endpoint of the size of the lesion that was significantly more marked in the heparan sulfate group than in the GAGPS group. Only a minority of patients in both groups used rescue paracetamol during the study. Both study drugs were well tolerated in terms of adverse events (no adverse events were reported with either drug). In conclusion, the results of the present study confirm that heparan sulfate 1% cream is a safe and effective local treatment for patients with haematomas and subcutaneous haematic extravasations of traumatic/surgical origin.

## Figures and Tables

**Table 1:** Demographic and baseline clinical characteristics. If not otherwise stated, entries are mean and 95% CI.

	Heparan sulfate	GAGPS
Demographic characteristics		
Gender (*n*-%):		
males	30 (41.7%)	21 (43.8%)
females	28 (58.3%)	27 (56.3%)
Age (years)	41.9 (37.4 to 46.5)	38.3 (34.0 to 42.6)
Weight (kg)	70.9 (67.5 to 74.4)	69.8 (66.7 to 72.9)
Height (cm)	172.4 (170.0 to 174.8)	172.5 (169.7 to 175.3)
BMI (cm/m^2^)	23.8 (22.9 to 24.7)	23.4 (22.6 to 24.3)

Baseline subjective symptoms and objective signs		
Pain at rest	1.3 (1.1 to 1.4)	1.2 (1.0 to 1.3)
Pain at movement	2.3 (2.1 to 2.4)	2.3 (2.2 to 2.5)
Functional disability	2.1 (2.0 to 2.3)	2.3 (2.1 to 2.5)
Oedema	2.4 (2.2 to 2.6)	2.5 (2.3 to 2.6)
Colour of the lesion	3.0 (3.0 to 3.0)	3.0 (2.9 to 3.0)
Total score	11.1 (10.6 to 11.6)	11.2 (10.8 to 11.7)

Size of haematomas/extravasation at baseline		
Minimum diameter (cm)	5.7 (5.1 to 6.3)	6.0 (5.4 to 6.7)
Maximum diameter (cm)	8.8 (8.1 to 9.6)	9.0 (8.1 to 9.9)
Min*Max diameter (cm^2^)	53.9 (44.4 to 63.4)	60.0 (44.0 to 76.1)

**Table:** (a)

Parameter	Mean decrease from baseline to endpoint		
	Mean [95% CI]		Differences between the adjusted means [95% CI]
	Heparan sulfate	GAGPS	
Pain at rest	−1.2 [−1.4 to −1.1]	−1.1 [−1.3 to −1.0]	0.02 [0.07 to 0.11]
Pain on movement	−1.9 [−2.0 to −1.7]	−1.4 [−1.6 to −1.1]	0.53 [0.27 to 0.78]
Functional disability	−1.6 [−1.8 to −1.4]	−1.3 [−1.5 to −1.1]	0.39 [0.09 to 0.68]
Oedema	−2.3 [−2.5 to −2.1]	−1.9 [−2.2 to −1.7]	0.41 [0.19 to 0.63]
Colour of the lesion	−2.8 [−2.9 to −2.7]	−2.1 [−2.3 to −2.0]	0.64 [0.42 to 0.87]

*Size of the lesion*			

Minimum diameter	−5.1 [−5.8 to −4.5]	−3.9 [−4.6 to −3.2]	1.48 [0.88 to 2.08]
Maximum diameter	−8.0 [−8.8 to −7.2]	−5.9 [−6.9 to −4.8]	2.25 [1.37 to 3.14]
Product of min*max diameter	−50.5 [−59.5 to −41.5]	−50.2 [−65.9 to −34.6]	5.95 [1.35 to 10.56]

**Table:** (b)

Parameter	Complete disappearance		
	*n* (%)		Fisher's exact test
	Heparan sulfate	GAGPS	

Pain at rest	46 (95.8)	45 (93.8)	NS
Pain on movement	30 (62.5)	11 (22.9)	<0.0001
Functional disability	30 (62.5)	15 (31.3)	0.0015
Oedema	43 (89.6)	28 (58.3)	<0.0001
Colour of the lesion	38 (79.2)	15 (31.3)	<0.0001
